# First regional reference database of northern Adriatic diatom transcriptomes

**DOI:** 10.1038/s41598-024-67043-4

**Published:** 2024-07-13

**Authors:** Mia Knjaz, Ana Baricevic, Mirta Smodlaka Tankovic, Natasa Kuzat, Ivan Vlasicek, Lana Grizancic, Ivan Podolsak, Martin Pfannkuchen, Tjasa Kogovsek, Daniela Maric Pfannkuchen

**Affiliations:** Center for Marine Research, Ruđer Bošković Institute, Rovinj, Croatia

**Keywords:** Computational biology and bioinformatics, Ecology, Molecular biology, Ocean sciences

## Abstract

Marine microbial communities form the basis for the functioning of marine ecosystems and the conservation of biodiversity. With the application of metagenomics and metatranscriptomics in marine environmental studies, significant progress has been made in analysing the functioning of microbial communities as a whole. These molecular techniques are highly dependent on reliable, well-characterised, comprehensive and taxonomically diverse sequenced reference transcriptomes of microbial organisms. Here we present a set of 12 individual transcriptome assemblies derived from 6 representative diatom species from the northern Adriatic Sea grown under 2 environmentally relevant growth conditions (phosphate replete vs. phosphate deprived). After filtering the reads and assembly, an average number of 64,932 transcripts per assembly was obtained, of which an average of 8856 were assigned to functionally known proteins. Of all assigned transcripts, an average of 6483 proteins were taxonomically assigned to diatoms (Bacillariophyta). On average, a higher number of assigned proteins was detected in the transcriptome assemblies of diatoms grown under replete media condition. On average, 50% of the mapped proteins were shared between the two growth conditions. All recorded proteins in the dataset were classified into 24 COG categories, with approximately 25% belonging to the unknown function and the remaining 75% belonging to all other categories. The resulting diatom reference database for the northern Adriatic, focussing on the response to nutrient limitation as characteristic for the region and predicted for the future world oceans, provides a valuable resource for analysing environmental metatranscriptome and metagenome data. Each northern Adriatic transcriptome can also be used by itself as a reference database for the (meta)transcriptomes and gene expression studies of the associated species that will be generated in the future.

## Introduction

Eukaryotic microbial communities are one of the key components for the functioning of marine ecosystems. Their combined metabolic activities drive a large range of oceanographic parameters, ecosystem functions and ecosystem services, they constitute the initial link in marine food webs, and their activity significantly contributes to biogeochemical nutrient cycling and oxygen production^1–4^. Members of these communities come from diverse eukaryotic lineages, present within each phylogenetic supergroup of eukaryotes^2^. Despite their ecological importance, much work remains to be done to understand the intricate ecological roles of these communities in marine environment. Diatoms (Bacillariophyta) represent an important group of marine eukaryotic microbial communities. In diverse marine environments diatoms are responsible for significant amounts of primary production and often, e.g. in the northern Adriatic, significantly contribute to the overall phytoplankton abundance as well^5–8^. Availability of nutrients and physiological response to nutrient limitation stand as the basic prerequisite for the survival, adaptation and success of diatoms in dynamic marine environments. Response of diatom species to nutrient stress have been extensively studied^9–20^ but still, diatom physiological responses to nutrient deficiency are poorly understood.

Application of high-throughput sequencing (omics) molecular techniques like metabarcoding, metagenomics and metatranscriptomics in marine environmental studies enabled significant progress in analysing the taxonomic, biological and functional diversity of microbial communities. The number of studies on the physiology and diversity of marine microbial communities using omics is increasing. Nevertheless, there is a lack of high-quality molecular resources needed for the complex analyses and interpretation of the generated (omics) data. These omics molecular techniques are highly dependent on the availability, coverage and curation of reference libraries or annotated sequence libraries as is the case for metatranscriptomics as well^21,22^. As far as the marine microbial community is concerned, eukaryotes in general and non–model organisms are particularly poorly represented among the available molecular omics resources^21,23–26^. Local, regional transcriptome datasets of these underrepresented microbial organisms form the core of successful omics data management, and are the base for any improvement in the resolution of environmental meta-omics analyses. Compared to other groups of marine microbial eukaryotes, many fully or partially sequenced and annotated genomes are available for diatoms^27–35^ or are being generated as part of ongoing sequencing projects (100 Diatom Genomes, https://jgi.doe.gov/csp-2021-100-diatom-genomes/). Nevertheless, available diatom genomes unsatisfactorily cover diatom diversity and genome sequencing still requires demanding efforts in its realization. Currently, reliable, well-characterised, comprehensive and taxonomically diverse sequenced reference transcriptomes stand as the most useful resource in metatranscriptomics analysis.

In 2014, Keeling et al. published a comprehensive database of the Marine Microbial Eukaryotic Transcriptome Sequencing Project (MMETSP) comprising 678 transcriptome assemblies from 405 species of marine microbial eukaryotes (including 71 diatom species) across various experimental conditions^23^. MMETSP database substantially expanded the available gene expression information of marine microbial eukaryotes and enhanced the understanding of the functional potential of diverse lineages of marine microbial eukaryotes, serving as the first molecular resource for some of these lineages^23^. In addition to the comprehensive MMETSP database, many individual studies of diatom transcriptomes have contributed significantly to the understanding of diatom physiology and related transcriptomes^20,30,36–44^. Specific regional transcriptome reference databases might allow to address regional peculiarities of transcripts and annotations and are expected to provide a higher functional and taxonomic resolution of the (regional) physiological patterns studied. At the same time, regional databases have global utility and significance for species-specific gene expression and biogeographic studies of those.

The northern Adriatic (NA) is a semi-enclosed, shallow coastal area of the Adriatic Sea and the northernmost part of the Mediterranean basin. The NA is exposed to a strong but fluctuating nutrient input, primarily from the Po River, one of the largest freshwater input in the entire Mediterranean^45^. The input from the Po River creates steep spatio-temporal ecological gradients in the northern Adriatic^17,18,46,47^ which together with the current circulation creates dynamic ecological conditions for the growth and succession of phytoplankton. In the NA, phytoplankton life strategies are mainly determined by light availability, temperature and the species' ability to cope with phosphorus limitation^17–19,48–51^. As the Po River is characterized by a much higher concentration of nitrogen, compared to phosphorus^52^ the species living and thriving in the northern Adriatic are used to unbalanced N/P ratio. Su et al. 2023., reported levels of alkaline phosphatase activity in the northern Adriatic to be the highest levels measured so far^53^. This is one of the central mechanisms to cope with phosphate limitation and one of the driving forces in the NA. The NA phytoplankton is largely dominated by diatoms, and species from the genera *Skeletonema*, *Pseudo-nitzschia* and *Chaetoceros* are known to be an important component of the NA phytoplankton community with some species occasionally forming very high abundances and toxic blooms^8,48,54–62^. The characterisation of the NA diatom community was mainly studied using classical light microscopy methods for species identification and physiological experiments^8,19,48,61,63^ while studies using high-throughput sequencing methods of molecular species identification and functional characterisation are still rare for the field^64–66^. Transcriptome and metatranscriptome studies of the diatom community are lacking for the NA.

Here, we present the first regional reference transcriptome database of diatom species isolated and cultivated from the northern Adriatic. A set of 12 individual transcriptome assemblies derived from 6 representative diatom species isolated from the northern Adriatic and grown under 2 experimental growth conditions (replete (F/2) vs. phosphorus deprived (P-limited)) was produced (Table 1). A detailed description of the creation, editing, curation and analysis of such a database is presented. To our knowledge, the reference transcriptome database created here is the first such valuable molecular ecology resource for the diatom community and, moreover, specific to the NA.

**Table 1:** Northern Adriatic (NA) dataset metadata.

Species	Culture ID	Sampling date	Station	Latitude	Longitude	Experimental condition
*Chaetoceros protuberans* Lauder, 1864	CIM827	05.11.2015	RV001	45°08N	13°61E	F/2
						P-limited
*Skeletonema marinoi* Sarno & Zingone 2005	CIM843	06.04.2016	SJ101	45°00N	12°83E	F/2
						P-limited
*Chaetoceros curvisetus* Cleve 1889	CIM950	19.11.2020	RV004	45°06N	13°55E	F/2
						P-limited
*Chaetoceros danicus* Cleve 1889	CIM964	11.12.2020	RV004	45°06N	13°55E	F/2
						P-limited
*Pseudo-nitzschia manni Amato & Montresor 2008*	CIM1008	23.7.2021	RV001	45°08N	13°61E	F/2
						P-limited
*Thalassiosira sp.Cleve, 1873*	CIM1063	22.12.2022	SJ101	45°00N	12°83E	F/2
						P-limited

## Materials and methods

### Sampling monoclonal cultures establishment

**Figure 1:**
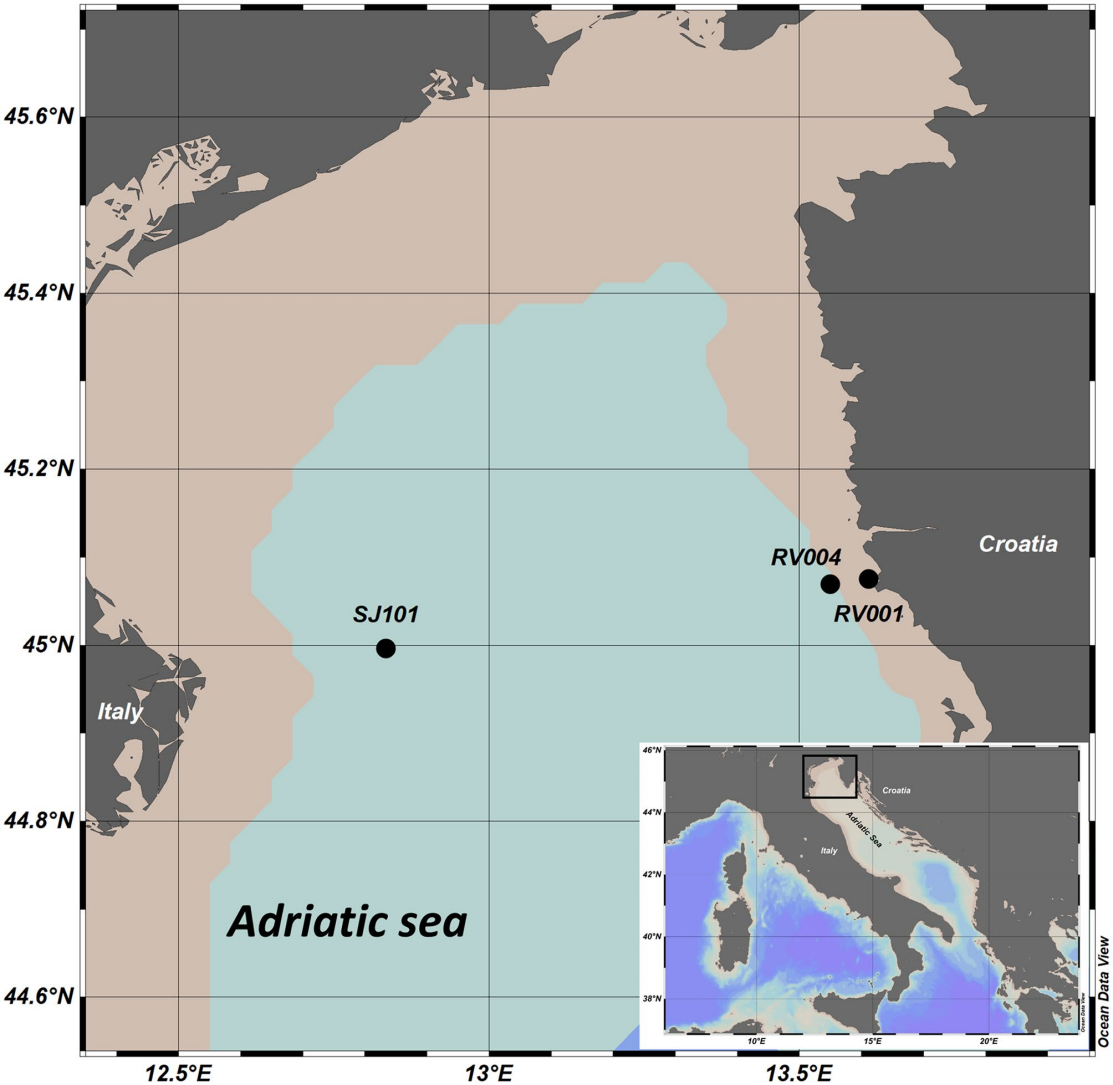
Sampling area. Stations RV001, RV004 and SJ101 where vertical net hauls for the isolation of the species were performed is marked.

Sampling for the isolation of monoclonal cultures was carried out at one coastal station (RV001) and two offshore stations (RV004, SJ101) in the northern Adriatic (Table 1, Fig. 1). At each station, vertical bottom—surface phytoplankton net (opening diameter 60 cm, length 2 m, mesh size 50 μm) hauls were performed. The diatom species were identified by light microscopy (Zeiss AxioObserver, Zeiss Oberkochen, Germany) and manually isolated from living net samples using Pasteur pipettes. Monoclonal cultures of the species were established by isolating single cells or single chain. Cells were grown in F/2 medium^67^, and incubated at 16 °C and 75 μmol photons m^−2^ s^−1^ in a 12:12 h light:dark photoperiod. The established monoclonal batch cultures were assigned to the phytoplankton cell culture collection of the Center for Marine Research (Table 1). Experimental in vitro incubations of the cultures were harvested for DNA extraction when the exponential growth phase was reached.

### Molecular species identification

**Figure 2:**
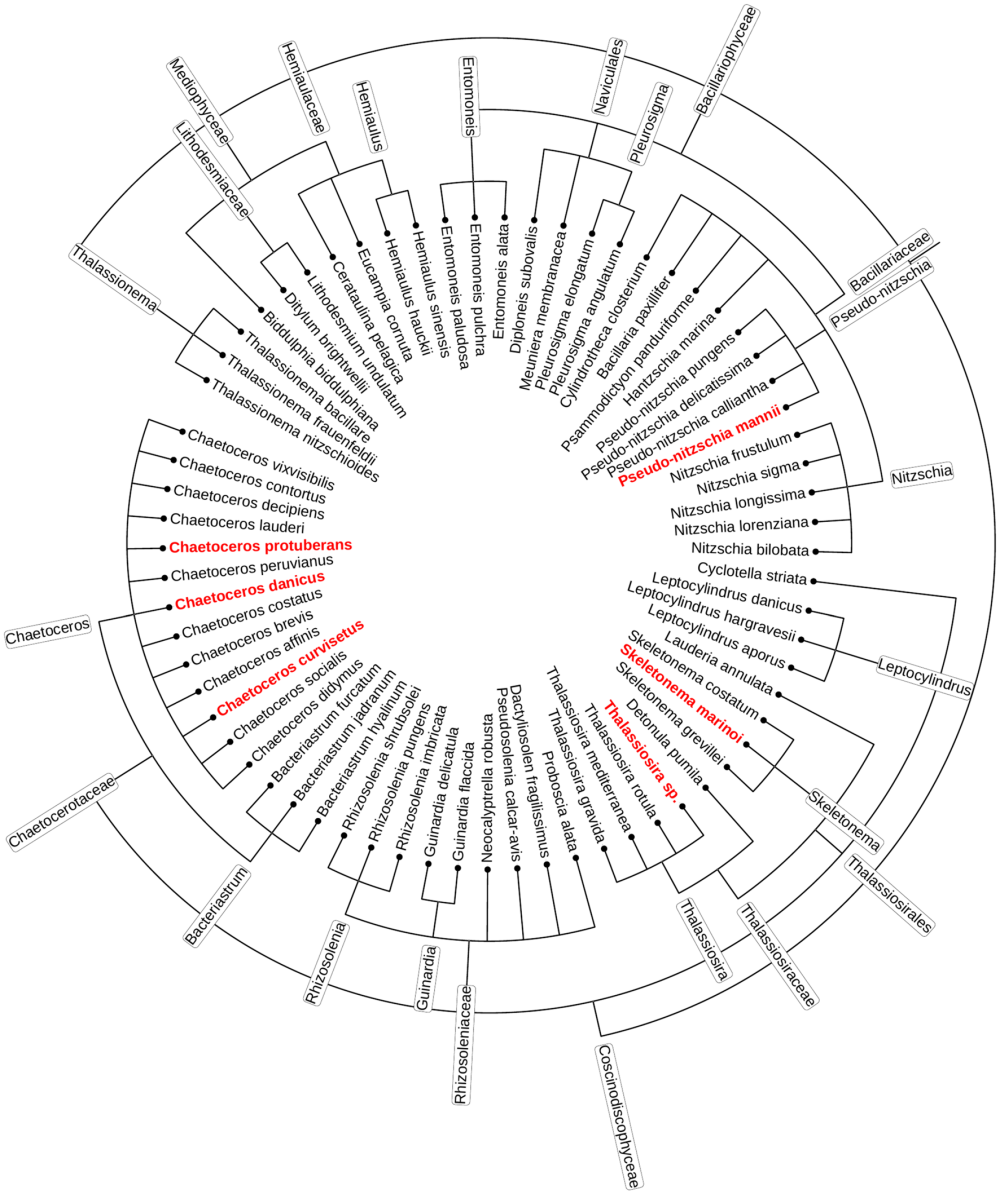
Most common diatom Adriatic plankton species phylogenetic tree. Species used in this study are indicated with red letters.

Three barcodes were used for the molecular species identification: V4 region of the small subunit (18S) ribosomal RNA gene, D1–D3 region of the large subunit (28S) ribosomal RNA gene and 5’end region of the ribulose 1,5-bisphosphate carboxylase large subunit (*rbcL*) (Supplementary Table 1). 30 mL of each cell culture was filtered on 1.2 µm cellulose filters (Merck Milipore) and frozen at − 80 °C until further processing. Genomic DNA was isolated using the DNeasy Plant Mini Kit (Qiagen) according to the manufacturer's instructions. PCR amplification was performed using DreamTaq DNA polymerase (Thermofisher Scientific). The reaction mixture (25 μL) contained 10 µL H_2_O, 12.5 µL DreamTaq Master Mix (2X), 0.75 µL of each primer (10 uM) and 1 µL genomic DNA (c ≤ 5 ng/µL). PCR reactions were performed in the SimpliAmp Thermal Cycler (Applied Biosystems). PCR conditions were as follows: an initial denaturation step of 5 min at 95 °C, 33 cycles of 40 s at 95 °C, 40 s at 52 °C and 1 min at 72 °C, and a final extension step of 5 min at 72 °C. The PCR-amplified products were purified using the NucleoSpin Gel and PCR clean-up mini kit (Macherey–Nagel) according to the manufacturer's instructions. The purified PCR products were sequenced at Macrogen Europe (The Netherlands). The barcoding sequences of the cultivated diatom species were stored in GenBank under the Accession Numbers: PP838189-PP838194, PP839066-PP839071 and PP839974-PP839978. Geneious software^68^ was used to align the sequences (from both ends) and further phylogenetic analyses were performed. BLAST was used for searching and comparison with the NCBI GenBank database^69,70^ (Fig. 2).

### In-vitro experimental incubations

Cultures for in-vitro experimental incubations were prepared as described in Smodlaka Tankovic et al.^19^. In brief, 2 mL of each established monoclonal batch culture (Table 1) were inoculated into 200 mL of the chosen growth medium (F/2 or P-limited). Nutrient-rich conditions (F/2) were simulated with medium F/2^67^. Dissolved inorganic P limitation stress (P-limited) was simulated using a P-limited medium (F/2 medium without added sodium hydrogen phosphate). Both growth media were prepared using seawater from the northern Adriatic, which was stored in the dark for 2 months and sterilized by double filtration through 0.22 μm pore size cellulose filter (Merck Millipore) and boiling in a microwave oven^71^. Prepared F/2 and P-limited cultures were incubated in climate chamber (Memmert ICH110, Germany) with a light–dark cycle of 12:12 h in sterile 250 mL vented culture flasks (easy flasks, Nunclon, Denmark) at 16 °C and irradiance of 75 μmol photons m^−2^ s^−1^. Cultures were incubated until the abundance of at least 10^5^ cells/L was reached for all cultures. At the end of incubation, 40–200 mL of each culture was filtered on 1.2 µm cellulose filters (Merck Millipore, Germany). Filtered culture volumes were chosen according to reached abundances, so that for the two growth conditions (F/2 and P-limited) of each culture the same number of cells was collected on the filter. Filters were stored at − 80 °C for the total RNA isolations.

### Isolation of the total RNA and transcriptome sequencing

Total RNA was isolated using a PureLink Mini kit (Invitrogen) with on-column Pure link DNase (Invitrogen) treatment, according to manufacturer instructions and adapted to filter isolations. Filters were immersed in 2.5 mL of lysis buffer and cells were removed from filter by pipetting, vortexing, and 5 min RT incubation. Lysate was centrifuged on 12,000×*g* for 5 min. Supernatants were transferred in clean falcon tubes and further processed in steps of RNA column binding, washing and elution. Total RNA was stored at − 80 °C until sent for transcriptome sequencing. Total RNA quality check, library preparations and sequencing were performed by Macrogen Europe (The Netherlands). RNA quality check was performed using Agilent 2200 TapeStation System (Agilent Technologies, United States). Library preparation and sequencing was conducted using TruSeq Stranded mRNA LT Sample Prep Kit and NovaSeq 6000 PE Illumina technology.

### Bioinformatics workflow

Paired-end reads (150 bp) were filtered for rRNA using SortMeRNA (v4.3.6) software^72^ with smr_v4.3_default_db.fasta as a reference and quality trimmed using Trimmomatic (v0.39)^73^ with settings for sliding window being 5:20 and minimum length of 50 bp. rRNA filtered and quality trimmed reads were assessed using FASTQC (v0.11.8) (https://www.bioinformatics.babraham.ac.uk/projects/fastqc/) and MULTIQC (v1.13) software (https://github.com/ewels/MultiQC). Transcripts were de novo assembled from filtered reads using Trinity software (v2.15.0)^74^ and default settings. Protein sequences were predicted using TransDecoder (v5.7.0) software (https://github.com/TransDecoder/TransDecoder) for open reading frame (ORF) prediction with default settings. Transcript quantification was conducted using Salmon software (v1.9.0)^75^ with index constructed for predicted protein sequences of each sample separately. Protein sequences with zero counts were filtered out and excluded from further analysis. Functional annotation of predicted protein sequences was performed using eggNOG-mapper^76^ via BioBam Omix box software (OmicsBox—Bioinformatics Made Easy, BioBam Bioinformatics, March 3, 2019, https://www.biobam.com/omicsbox) for eggNOG^77^, KEGG^78^ and GO^79^ database annotations. Only sequences reaching both e-value and bitscore thresholds of 1 × 10^–5^ and 50, respectively, were considered successfully annotated.

eggNOG v 5.0 (Evolutionary genealogy of genes: Non-supervised orthologous groups), KEGG (Kyoto encyclopedia of genes and genomes) and GO (Gene ontology) are publicly available databases that classify genes into orthologues groups (OGs) and provide functional descriptions. GO classifies OGs into three broader functional categories; molecular function (MF), cellular component (CC) and biological process (BP)^80^. KEGG classifies OGs into broader functional categories in the context of KEGG molecular networks, namely, pathway maps, BRITE hierarchies and modules^81^ while eggNOG uses COG database broader functional categories system^82^.

Via eggNOG mapper algorithm for taxonomy prediction we assigned proteins their taxonomic origin. Only protein sequences corresponding to taxonomic level Bacillariophyta were used for the functional annotation analyses. Of all the Bacillariophyta OGs in the eggNOG database, 70% are functionally annotated, while the remaining 30% represent diatom OGs with unknown functions. Thus, functional annotation via eggNOG-mapper facilitates the distinction between diatom proteins with known functions and those with unknown functions, i.e., proteins identified in diatom genomes but whose functions are yet to be determined. To assign protein to a broader functional category a COG functional categories system was used^82^.

In order to compare our transcriptomes with the transcriptomes of the same species in the MMETSP database, we used files generated in Johnson et al.^83^. Seven *Skeletonema marinoi* transcriptomes (sample IDs: MMETSP0319, MMETSP0320, MMETSP0918, MMETSP0920, MMETSP1039, MMETSP1040, MMETSP1428) and four *Chaetoceros curvisetus* transcriptomes (sample IDs: MMETSP0716, MMETSP0717, MMETSP0718, MMETSP0719) from different growth conditions were found in MMETSP dataset. We downloaded predicted protein sequences (https://zenodo.org/records/257026), quantification files (https://zenodo.org/records/257145) and contig ID name maps (https://zenodo.org/records/3247846). Transcriptomes metadata was found in NCBI Biosample database for Bioproject accession number PRJNA231566 (Table 2). Similar to our dataset, MMETSP predicted protein sequences were generated using TransDecoder software and quantification files using Salmon software. Protein sequences were functionally annotated following the same bioinformatic workflow as for NA transcriptomes, using eggNOG-mapper. Proteins corresponded to transcripts with zero counts were filtered from the dataset. Transcriptomes under sample IDs MMETSP0716 and MMETSP0717 were excluded from analysis due to insufficient number of predicted proteins (6) and missing quantification file.

**Table 2:** MMETSP dataset metadata.

Sample name	Species	Geographic region	Condition
MMETSP0319	*Skeletonema marinoi*	Baltic Sea	F/2 (-Si -Cu)
MMETSP0320	*Skeletonema marinoi*	North Sea	F/2
MMETSP0918	*Skeletonema marinoi*	Atlantic Ocean	F/2
MMETSP0920	*Skeletonema marinoi*	Atlantic Ocean	F2—Si
MMETSP1039	*Skeletonema marinoi*	Adriatic Sea	F/2—light
MMETSP1040	*Skeletonema marinoi*	Adriatic Sea	F/2 + light
MMETSP1428	*Skeletonema marinoi*	Pacific Ocean	Standard Aquil
MMETSP0718	*Chaetoceros curvisetus*	Pacific Ocean	ASW—NO3
MMETSP0719	*Chaetoceros curvisetus*	Pacific Ocean	ASW + nocodazole

## Results

### Dataset structure

Transcriptome sequencing of six diatom species grown in two phosphate conditions (F/2 and P-limited) resulted in a dataset that consisted of on average 43,261,377 reads/sample (Table 3). After rRNA filtering, on average 31,334,595 mRNA reads/ sample remained (Supplementary Table 2). Additionally, approximately 2% of poor-quality mRNA reads were removed from each sample through Trimmomatic quality trimming (Supplementary Table 2). Overall, after pre-processing steps the dataset consisted of 365,178,904 reads (average 30,431,575 reads/sample) (Table 2). Trinity de novo assembly constructed 779,184 transcripts (on average 64,932 transcripts/sample) with an average length of 731 bp (Table 3). Obtained transcripts coded for 422,974 predicted protein sequences (on average 35,248 proteins/sample) (Supplementary Table 3). When proteins corresponding to transcripts with zero counts (erroneous or redundant transcripts) were filtered out from the dataset, the finalized dataset of the northern Adriatic diatom transcriptomes consisted of 106,274 protein sequences (average 8,856 protein sequences/sample) (Table 3).

**Table 3:** Overview of the NA transcriptome final dataset characteristics (sequences abundance and length) through bioinformatics processing steps for each diatom culture and growth condition.

Culture ID	Species	Condition	Number of reads	Number of filtered reads	Number of transcripts	Average transcript length (bp)	Number of protein sequences*
CIM827	*Chaetoceros protuberans*	F/2	43,869,248	35,892,984	4204	971	954
		P-limited	46,060,370	19,291,546	55,259	561	6,623
CIM843	*Skeletonema marinoi*	F/2	47,142,298	43,905,102	64,544	1011	16,193
		P-limited	46,959,304	36,962,568	70,298	709	10,695
CIM950	*Chaetoceros curvisetus*	F/2	45,113,058	34,955,780	50,374	898	9,715
		P-limited	35,562,540	7,855,370	22,886	350	954
CIM964	*Chaetoceros danicus*	F/2	41,065,412	36,587,302	75,087	917	12,112
		P-limited	35,182,380	23,446,778	63,106	866	8551
CIM1008	*Pseudo-nitzschia mannii*	F/2	46,749,028	36,708,516	52,978	687	8235
		P-limited	43,417,630	16,183,690	34,986	505	3244
CIM1063	*Thalassiosira* sp.	F/2	38,814,556	29,241,382	108,010	492	5677
		P-limited	49,200,702	44,147,886	177,452	807	23,321

*Number of protein sequences corresponds to proteins remained after the exclusion of proteins corresponding to transcripts with zero counts.

### Dataset analysis

**Figure 3:**
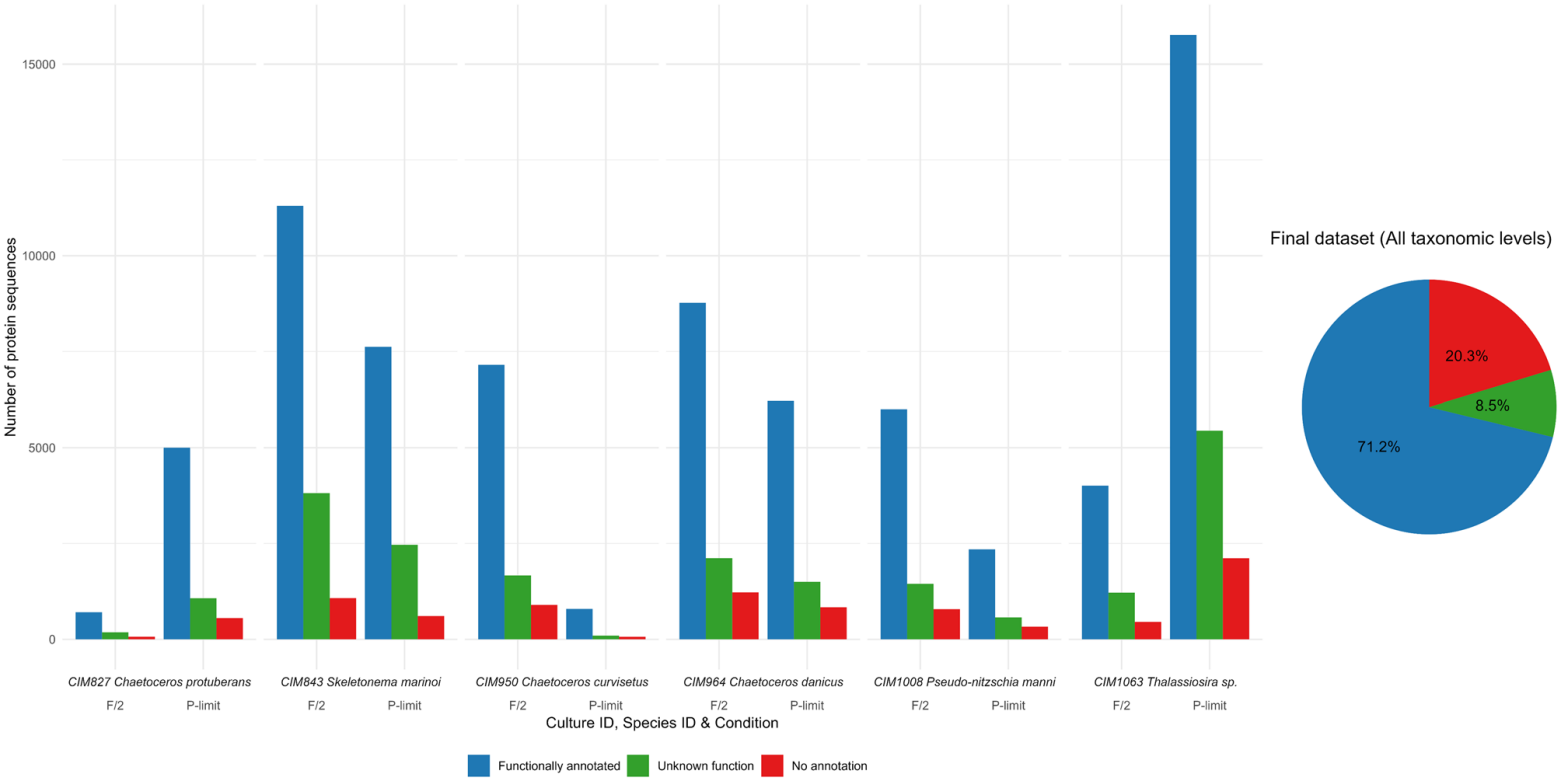
Representation of functionally annotated (blue), function unknown (orange) and protein sequences with no annotations. Piechart represents percentages of protein sequences corresponding to each annotation level (group) in the final dataset. Barchart represents total number or protein sequences in each sample of the final dataset separately.

To further analyse the final dataset, the protein sequences were functionally annotated using eggNOG-mapper software to obtain the eggNOG description, GO and/or KO terms for each protein sequence as well as taxonomic affiliation. Using eggNOG-mapper and after reaching the e-value and bitscore thresholds (see “Methods”), 97,271 protein or 91.5% of all protein sequences in the final dataset could be successfully annotated by using the searched databases (on average 8106 protein sequences/ sample). For 9003 or the remaining 0.5% of all protein sequences (750 protein sequences/sample) we haven’t found annotation passing the given e-value and bitscore tresholds (Fig. 3). After functional annotation with eggNOG- mapper, successfully annotated protein sequences could be divided in two categories, those that received at least one functional annotation and those corresponding to proteins with an unknown function. In total, 75,682 protein sequences (on average 6307 proteins/sample) received at least one functional annotation and 21,589 protein sequences (on average 1799 protein sequences/sample) are corresponding to proteins of unknown function (Fig. 3). Most protein sequences of the final dataset were assigned an eggNOG description (77.3%). Approximately half of proteins (51.5%), were not only assigned an eggNOG description but also associated with a KO or GO term or both. Notably, many protein sequences annotated with KO or GO received more than one term, while all eggNOG annotated protein sequences received only one eggNOG description. 34% of KO annotated protein sequences received more than one KO term, while 96.4% of all GO annotated protein sequences received more than one GO term. Overall, a total of 75,188 protein sequences were assigned an eggNOG description, 47,987 were assigned a KO term, and 30,081 were assigned a GO term (Supplementary Table 3).

The highest number of annotated protein sequences (referred to as proteins in the following text) was found in the CIM1063 (*Thalassiosira* sp.) culture grown under P-limited conditions, while the CIM827 (*C. protuberans*) culture grown in F/2 had the lowest number of annotated proteins. For most cultures, a higher number of annotated proteins was associated with F/2 growth conditions. Only CIM827 (*C. protuberans*) and CIM1063 (*Thalassiosira* sp.) had more annotated proteins in P-limited condition (Fig. 3). The highest number of proteins received functional annotation based on eggNOG description (on average 6266 proteins/sample), followed by KO annotations (on average 3999 proteins/sample) and GO annotations (on average 2507 proteins/sample) (Supplementary Table 3).

**Figure 4:**
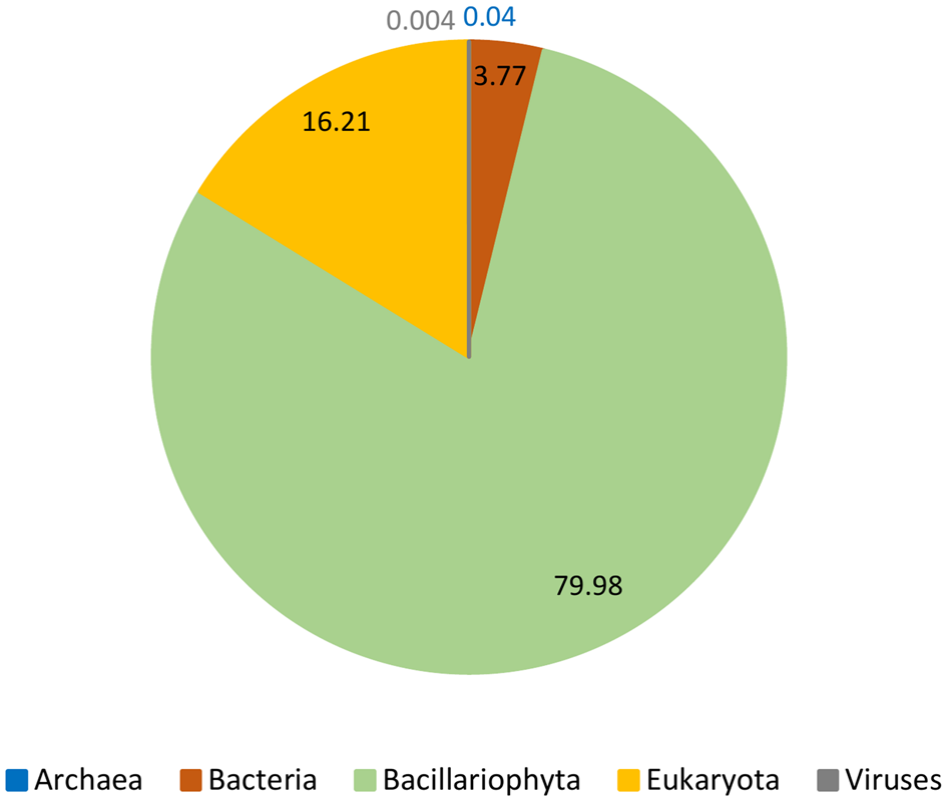
Proportion of annotated proteins assigned to different taxonomic groups/levels (Bacillariophyta, Eukaryota, Bacteria, Archea and Viruses).

Since NA diatom cultures used for experimental incubations and transcriptome sequencing were not axenic cultures, a part (on average 20% proteins/sample) of annotated protein sequences was assigned to taxonomic groups other than diatoms (Bacillariophyta). The number of non-diatom annotated proteins didn’t notably differ between the cultures or the two growth conditions. The majority of non-diatom annotated proteins in the final dataset were assigned to Eukaryota (15,764 proteins) and Bacteria (3666). The Eukaryota group included transcriptome sequences of eukaryotic taxa (e.g. heterotrophic protists) that do not belong to the Bacillariophyta, as well as sequences that were classified as Eukaryota at the lowest taxonomic level. In total, around 80% of all annotated protein sequences corresponded to diatoms (Bacillariophyta) and the remaining 20% to Eukaryota (16%), Bacteria (around 4%) and Archaea (less than 1%) (Fig. 4). The only exception was sample CIM950 P-limited (*C. curvisetus*) that had more proteins assigned to Bacteria than to Eukaryota, 14% and 9%, respectively. Protein sequences annotated as Bacillariophyta will be referred to as the diatom dataset in subsequent text.

It total, the diatom dataset contains 77,796 sequences (on average 6483 proteins/sample). In this dataset 58,714 proteins (on average 4893 proteins/sample) received at least one functional annotation and 19,082 proteins (on average 1590 proteins/sample) are corresponding to diatom proteins of unknown function. Most protein sequences of the diatom dataset were assigned an eggNOG description (around 75%) (Supplementary Table 4). The majority of these proteins (approximately 68%), were not only assigned an eggNOG description but also associated with a KO or GO term or both. Also, most sequences were assigned more than one KO and GO term. 34.9% of KO annotated sequences received more than one KO term, while 96.7% of all GO annotated sequences received more than one GO term. Overall, a total of 58,295 protein sequences in the diatom dataset were assigned an eggNOG description, 38,453 were assigned a KO term, and 25,470 were assigned a GO term (Supplementary Table 4).

### Diatom dataset analysis (Functional analysis)

**Figure 5:**
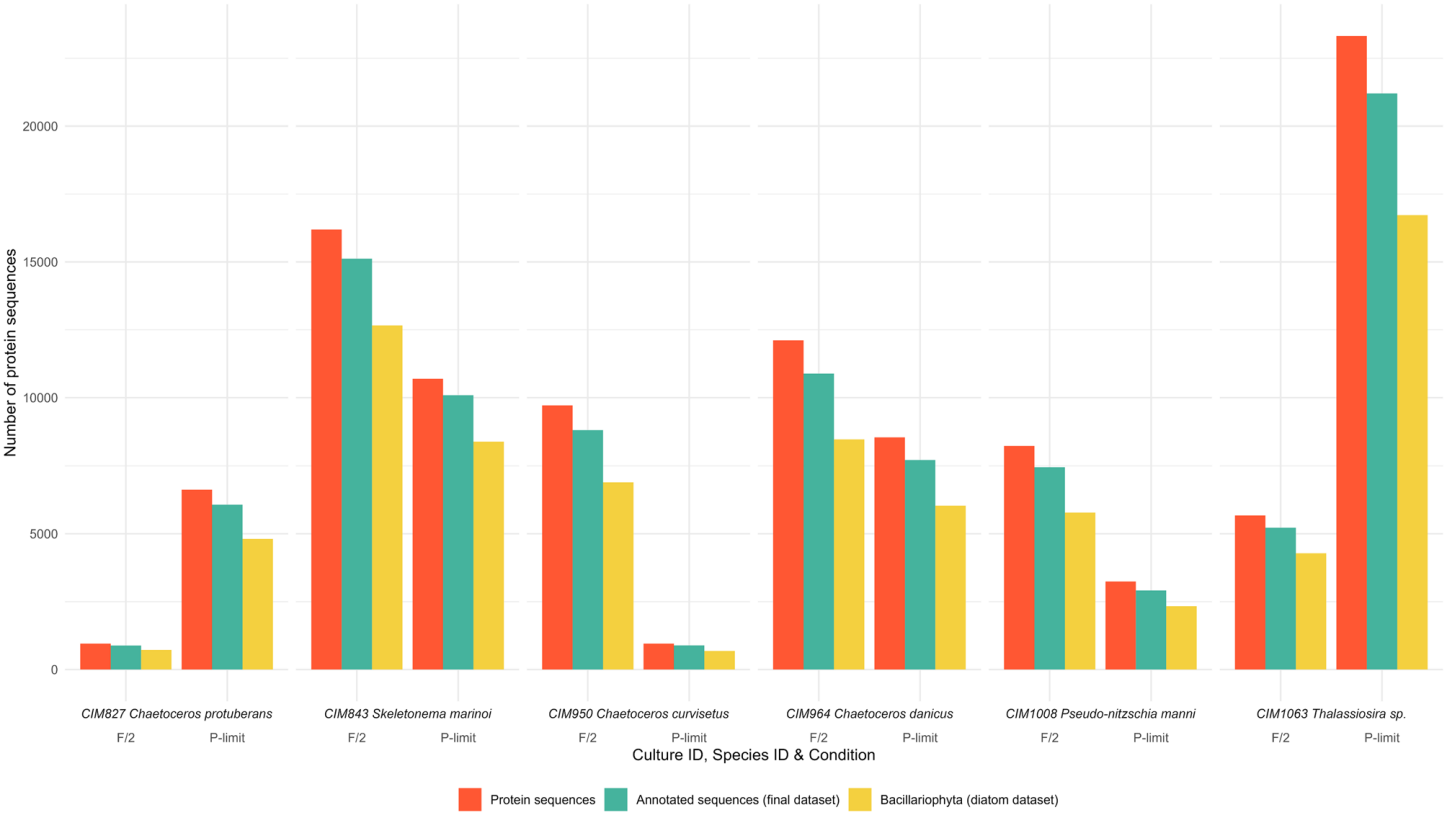
Representation of annotated protein sequences in each culture and growth condition after functional annotation of the NA transcriptome dataset: total number of protein sequences (green), successfully annotated sequences (passing e-value and bitscore threshold)—final dataset (yellow) and successfully annotated protein sequences assigned to best taxonomic level Bacillariophyta—diatom dataset (blue).

To further analyse the final dataset according to functional annotation, only diatom assigned protein sequences were used (the diatom dataset) (Fig. 5). Unique annotations were obtained when protein sequences of the diatom dataset were merged according to identical eggNOG description/KO term/GO term. All that resulted in 21,458 proteins with unique annotationbased on eggNOG description, 21,926 based on KO term and 16,524 based on GO term (Supplementary Table 4). The highest number of unique annotations was found in CIM1063 (*Thalassiosira* sp.) culture grown under P-limited conditions, while the CIM827 (*C. protuberans*) culture grown in F/2 had the lowest number of unigenes (Supplementary Table 4). For most cultures, the higher number of unique annotations were associated with F/2 growth condition (Supplementary Table 4). Only CIM827 (*C. protuberans*) and CIM1063 (*Thalassiosira* sp.) had more unique annotations in the P-limited condition (Supplementary Table 4). For most cultures, merging the identical KO term gave the highest number of unique annotations (on average 1827 unique annotations/sample) (Supplementary Table 4). Following was the unique annotations obtained by merging the identical eggNOG description (1788 unique annotations/sample) (Supplementary Table 4). Only cultures CIM827 (*C. protuberans*) and CIM1063 (*Thalassiosira* sp.) grown under F/2 conditions and cultures CIM950 (*C. curvisetus*) and CIM1008 (*P. mannii*) grown under P-limited conditions had more unique annotations based on eggNOG description than on KO term (Supplementary Table 4).

**Figure 6:**
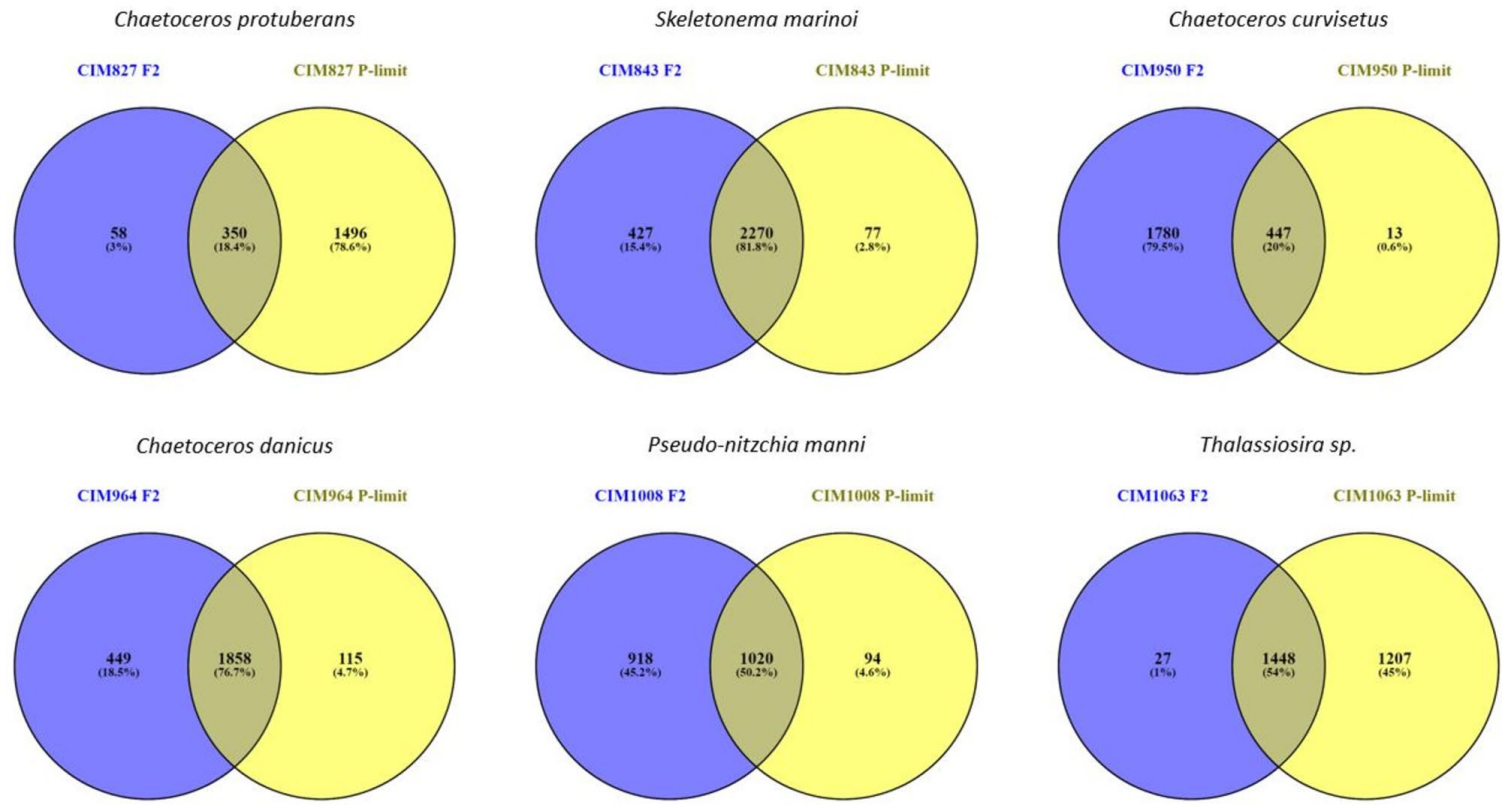
Comparison of the eggNOG annotated protein structure in diatom dataset between different cultures and growth conditions.

To further analyse the diatom dataset, we used unique annotations obtained by merging the unique eggNOG annotations. On average, 50% of unique annotations were shared between cultures grown under different conditions (F/2 and P-limited) while 27.1% and 22.7% were specific to F/2 and P-limited, respectively (Fig. 6). Cultures CIM827 (*C. protuberans*) and CIM1063 (*Thalassiosira* sp*.*) were characterised by a notably higher number of unique annotations (1496 and 1207 respectively) found only in P-limited cultures. In contrast, the number of unique annotations specific for F/2 was notably higher (1780) in culture CIM950 (*C. curvisetus*) than in the other cultures (Fig. 6). For culture CIM843 (*S. marinoi*), the highest number of shared unique annotations (2270) was found between the two growth conditions (Fig. 6). Overall, 222 unique annotations were shared among all diatom species under F/2 conditions, and 255 unique annotations were shared under P-limited conditions, respectively.

**Figure 7:**
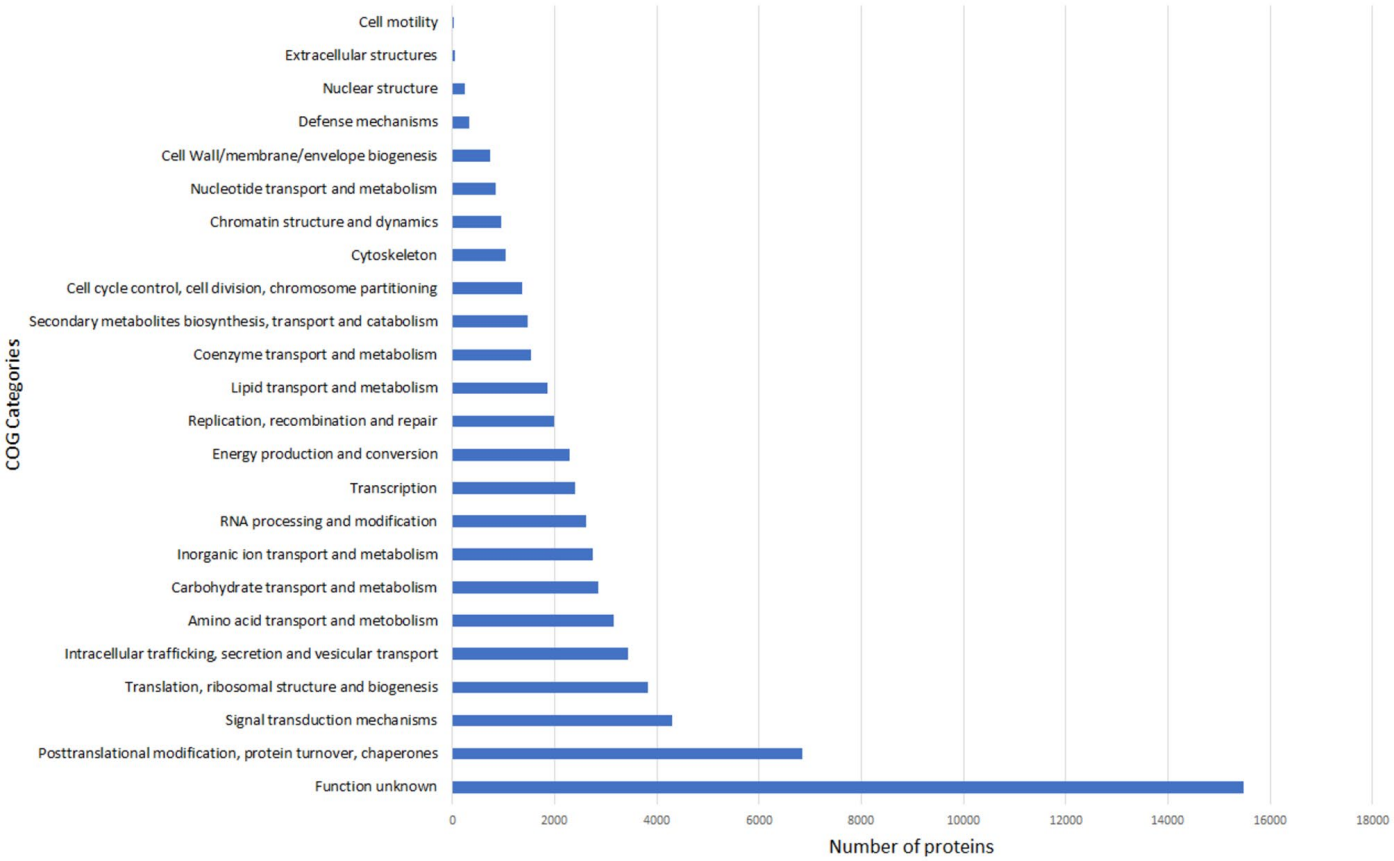
Representation of COG categories distrubution in diatom dataset across all cultures. x-axis—number of all detected proteins that passed e-value and bitscore treshold and were assigned to Best taxonomic level Bacillariophyta. y-axis—COG categories foun in diatom dataset. (Out of 26 COG categories 24 were found, missing functions: General function prediction only and Mobilome: prophages, transposons).

COG categories^82^ were utilized to assign proteins to broader functional categories. Diatom proteins annotated with eggNOG description were classified into 24 out of 26 possible COG categories (Fig. 7). Across most cultures, the "function unknown" category consistently had the highest number of proteins, followed by the "posttranslational modification, protein turnover, chaperones" category and the "signal transduction mechanisms" category. Notably, only culture CIM950 P-limited (*C. curvisetus*) exhibited the highest protein count associated with the "Translation, ribosomal structure and biogenesis" category.

### MMETSP database comparison

To compare the results with diatom transcriptome datasets available in the MMETSP database, analysis of the MMETSP diatom transcriptome datasets was conducted according to the pipeline for transcriptome analysis applied for the NA transcriptomes (described in Methods).

De novo assembly constructed on average 31,878 transcripts/sample for *S. marinoi* MMETSP dataset and on average 8816 transcripts/sample for *C. curvisetus* MMETSP dataset with average transcript length of 815 and 567 bp, respectively (Table 4). In comparison, *S. marinoi* NA transcriptomes had on average 36,630 transcripts/sample with an average length of 860 bp while *C. curvisetus* NA transcriptomes had on average 67,421 transcripts/sample with an average length of 624 bp (Table 4). Based on the number of assembled transcripts, *S. marinoi* MMETSP and NA transcriptomes were not very different. The average number of assembled transcripts for MMETSP *S. marinoi* transcriptomes was 31,878 transcripts/sample and NA *S. marinoi* transcriptomes had 50,374 (CIM843 F/2) and 22,886 (CIM843 P-limited) transcripts assembled (Table 4). The length of assembled transcripts varied from 606 (MMETSP0920) to 1011 (CIM843 F/2) with the average length being 825 bp (Table 4). The number of assembled transcripts in *C. curvisetus* transcriptomes however, did differ. The average number of assembled transcripts of *C. curvisetus* MMETSP transcriptomes was 8816 and for *C. curvisetus* NA transcriptomes, 67,421 (Table 4). The length of the transcripts varied from 350 (CIM950 P-limited) to 652 (MMETSP0718) (Table 4). The number of predicted protein sequences also varied in all cultures. Based on predicted protein sequence numbers, there is no notable difference between the NA and MMETSP transcriptomes. For obtained transcripts an average of 14,539 protein sequences/sample was predicted in *S. marinoi* MMETSP dataset and an average of 4160 protein sequences/sample in *C. curvisetus* MMETSP dataset. In comparison, the NA dataset had on average 13,444 predicted protein sequences/sample for *S. marinoi* transcriptomes and 5335 predicted protein sequences/sample for *C. curvisetus* transcriptomes (Table 4). These numbers applied to number of protein sequences after the elimination of those corresponding to transcripts with zero counts. *S. marinoi* transcriptome with the highest amount of predicted protein sequences (20,681) was MMETSP0918 generated in F/2 condition (Table 4). NA *S. marinoi* transcriptome generated in F/2 condition (CIM843 F/2) had 16,193 predicted protein sequences (Table 4). The *C. curvisetus* transcriptome with the highest number of predicted proteins was CIM950 F/2 with 9715 predicted protein sequences followed by MMETSP0718 (ASW -NO_3_) with 4934 predicted protein sequences (Table 4). Overall, the transcriptome from *S. marinoi* generated in F/2 -Si condition had the lowest number of predicted protein sequences, only 444. Despite this number being notably different from other *S. marinoi* transcriptomes, it appears that the average number of predicted protein sequences varies more between the two species than within different conditions for the same species. Specifically, the difference is notable in the number of predicted proteins being one order of magnitude greater for *S. marinoi* compared to *C. curvisetus*.

**Table 4:** General structure comparison between MMETSP and NA transcriptomes of *S. marinoi* and *C. curvisetus*. * Number of protein sequences corresponds to proteins remained after the exclusion of proteins corresponding to transcripts with zero counts.

Culture ID	Species	Condition	Geographic region	Trinity		TransDecoder	eggNOG- mapper
				Number of transcripts	Average transcript length	Number of predicted protein sequences*	Number of annotated protein sequences
MMETSP0319	*Skeletonema marinoi*	F/2 (-Si -Cu)	Baltic sea	33,153	962	18,699	16,898
MMETSP0320	*Skeletonema marinoi*	F/2	North Sea	34,068	950	19,301	17,561
MMETSP0918	*Skeletonema marinoi*	F/2	Atlantic Ocean	32,428	778	20,681	18,546
MMETSP0920	*Skeletonema marinoi*	F/2 -Si	Atlantic Ocean	33,291	606	444	413
MMETSP1039	*Skeletonema marinoi*	F/2 -light	Adriatic Sea	30,075	832	11,383	10,263
MMETSP1040	*Skeletonema marinoi*	F/2 + light	Adriatic Sea	26,989	820	11,209	10,079
MMETSP1428	*Skeletonema marinoi*	Standard Aquil	Pacific Ocean	33,140	757	20,054	18,172
CIM843	*Skeletonema marinoi*	F/2	Adriatic Sea	64,544	1011	16,193	15,118
CIM843	*Skeletonema marinoi*	P-limit	Adriatic Sea	70,298	709	10,695	10,089
MMETSP0718	*Chaetoceros curvisetus*	ASW -NO3	Pacific Ocean	9747	652	4934	4260
MMETSP0719	*Chaetoceros curvisetus*	ASW + nocodazole	Pacific Ocean	7884	482	3385	3322
CIM950	*Chaetoceros curvisetus*	F/2	Adriatic Sea	50,374	898	9715	8817
CIM950	*Chaetoceros curvisetus*	P-limit	Adriatic Sea	22,886	350	954	889

After functional annotations with eggNOG mapper, the percentage of protein sequences with annotations passing e-value and bitscore thresholds (see “Methods”) also didn’t differ greatly when comparing MMETSP and NA transcriptomes. For almost all samples it exceeded 90%. The percentage of protein sequences that received at least one functional annotation (eggNOG description, KO, GO) was 75% on average for MMESTP and NA transcriptomes of *S. marinoi*. The remaining 25% corresponded to proteins of unknown function (Table 5). For *C. curvisetus* MMETSP and NA transcriptomes the average percentage of protein sequences that received at least one functional annotation was 89% and 85%, respectively (Table 5). The remaining percentage of proteins with unknown function was smaller than in *S. marinoi*, 11% and 15% respectively (Table 5). Similar to NA transcriptomes, the highest number of annotations per sample was based on eggNOG description following by KO and GO term (Table 5). In both MMETSP and NA transcriptomes 99% of proteins with at least one functional annotation received an eggNOG description, 59–80% received a KO term and 21–55% received a GO term (Table 5).

**Table 5:** Comparison of the MMETSP *S. marinoi* and *C. curvisetus dataset* and NA dataset after eggNOG functional annotation.

Culture ID	Species	Condition	Any functional annotation	Unknown function	eggNOG description	KOs	GOs
MMETSP0319	*Skeletonema marinoi*	F/2 (-Si -Cu)	12,766	4132	12,680	7671	4349
MMETSP0320	*Skeletonema marinoi*	F/2	13,204	4601	13,105	7841	4413
MMETSP0918	*Skeletonema marinoi*	F/2	13,987	4559	13,876	8405	4476
MMETSP0920	*Skeletonema marinoi*	F/2 -Si	309	104	306	198	108
MMETSP1039	*Skeletonema marinoi*	F/2 -light	7680	2583	7619	4733	2699
MMETSP1040	*Skeletonema marinoi*	F/2 + light	7499	2580	7439	4687	2676
MMETSP1428	*Skeletonema marinoi*	Standard Aquil	13,760	4412	13,671	8439	4578
CIM843	*Skeletonema marinoi*	F/2	11,304	3814	11,214	6779	4183
CIM843	*Skeletonema marinoi*	P-limited	7624	2465	7561	4752	2987
MMETSP0718	*Chaetoceros curvisetus*	ASW -NO3	3569	691	3560	2464	1477
MMETSP0719	*Chaetoceros curvisetus*	ASW + nocodazole	3150	172	3149	2517	678
CIM950	*Chaetoceros curvisetus*	F/2	7152	1665	7112	4770	3043
CIM950	*Chaetoceros curvisetus*	P-limited	792	97	790	639	443

Any functional annotation—number of protein sequences with at least one annotation (eggNOG description, KO or GO term) passing e-value and bitscore tresholds. Unknown function—number of protein sequences passing an e-value and bitscore tresholds corresponding to proteins of unknown function. eggNOG description—number of protein sequences annotated with eggNOG description. Kos—number of protein sequences annotated with KO term. GOs—number of protein sequences annotated with GO term.

**Figure 8:**
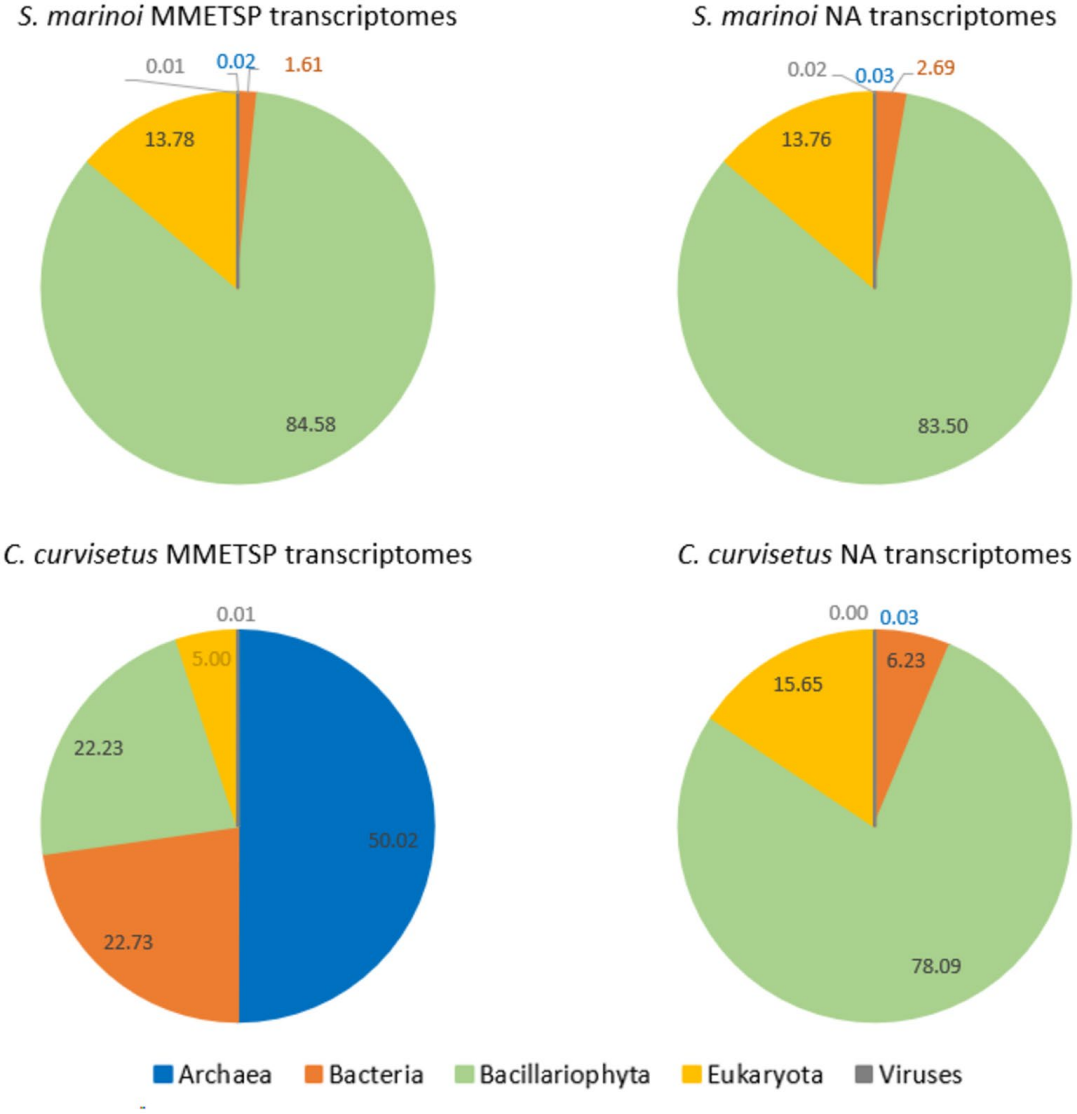
Comparison of proportions of annotated proteins assigned to different taxonomic groups/levels (Bacillariophyta, Eukaryota, Bacteria, Archea and Viruses) in *S. marinoi* and *C. curvisetus* MMETSP and NA transcriptomes.

We applied eggNOG best taxonomic level prediction algorithm to assign best taxonomic levels to predicted protein sequences. *S. marinoi* transcriptomes from both MMETSP and NA datasets had 83–85% of proteins assigned to Bacillariophyta (diatoms), 12–14% assigned to Eukaryota, 1–3% assigned to Bacteria and less than 1% assigned to Archaea and Viruses (Fig. 8). Best taxonomic levels of *C. curvisetus* transcriptomes for both datasets had slightly less amount of diatom proteins, 76–78% for samples MMETSP0718, CIM950 F/2 and CIM950 P-limited. These results are quite consistent with all NA transcriptomes with diatom proteins ranging from 77 to 84% per sample. Transcriptome MMETSP0719 had the lowest amount of diatom proteins, only 22%. The highest amount of proteins in that sample corresponded to best taxonomic level Archaea. This sample was treated with nocodazone, a known microtubule inhibitor that might have a negative effect on diatom growth and metabolic activity.

To further analyse *S. marinoi* and *C. curvisetus* MMETSP and NA datasets according to functional annotation, only diatom assigned protein sequences were utilized. Unique annotations were obtained when protein sequences of the diatom dataset were merged according to identical eggNOG description/KO term/GO term. The highest amount of unique annotations for most cultures was reached by merging the unique KO term, followed by eggNOG and GO unique annotations (Supplementary Table 5). The highest number of unique annotations was found in *S. marinoi* transcriptome MMETSP1428, 2731 eggNOG, 2916 KO and 2021 GO unique annotations (Supplementary Table 5). The transcriptome with the lowest amount of unique annotations was *C. curvisetus* MMETSP0719 with the lowest number of diatoms assigned proteins (nocodazone treatment) (Supplementary Table 5). The average number of unique annotations per sample for *S. marinoi* MMETSP transcriptomes was 2131 eggNOG, 2220 KO and 1513 GO unique annotations (Supplementary Table 5). The average number of unique annotations per sample for *S. marinoi* NA transcriptomes was 2523 eggNOG, 2649 KO and 2003 GO unique annotations (Supplementary Table 5). *C. curvisetus* transcriptomes CIM950 P-limited and MMETSP0718 had numbers of unique annotations comparable with those of *S. marinoi* while CIM950 P-limited and MMETSP0719 had notably lower number of unique annotations (Supplementary Table 5).

Diatom proteins that received an eggNOG description were classified into 24 out of 26 possible COG categories. The majority of diatom proteins were assigned an "Unknown function" after COG categorization. Subsequently, in most cultures, the categories with the highest representation after "Unknown function" were "Posttranslational modification, protein turnover, chaperones" and "Signal transduction mechanisms” (Fig. 7). The COG functional categories did not reveal any patterns of expression considering condition or species.

## Discussion

The raw data obtained in this study consisted of 519,136,526 strand-specific pair-end Illumina reads for 12 individual RNA samples generated from northern Adriatic diatom cultures grown in two conditions (Table 3). The condition of P-limited media was indicative of the typical environmental parameters of the northern Adriatic since the life strategies of phytoplankton in the northern Adriatic are significantly influenced by the limitation of phosphorus^17,18,49^. The applied bioinformatic pipeline resulted in a finalised dataset of the northern Adriatic diatom transcriptomes consisting of 106,274 protein sequences with an average of 8856 protein sequences per sample (Table 3). To inspect the dataset, eggNOG-mapper was applied to assign functional and taxonomic annotation to protein sequences. In total, 97,271 proteins or 91.5% of the proteins in final dataset were successfully annotated (Fig. 3). Distinguishing proteins with annotations revealed two categories: those receiving at least one functional annotation (eggNOG description, KO, or GO term) and those with unknown function but previously identified in sequenced genomes. In total, around 78% of all annotated proteins fall into the first category, while the remaining 22% fall into the second category (Fig. 3). Out of all annotated protein sequences, 77% were assigned an eggNOG description, while KO and GO term were assigned to 49% and 31% of annotated protein sequences, respectively (Supplementary Table 3). This distribution aligns with expectations, considering that the eggNOG database (v.5) is the most comprehensive, with 4.4 million orthologous groups (OGs) distributed across 379 taxonomic levels^77^. In characterising the NA reference database, the aim was to highlight all three annotation methods, since the use of new omics data is still quite rare and represents a new research approach. Therefore, detailed annotation at this stage would allow for more constructive and easier improvements and/or comparisons of omics research in the future. Working with a single unique annotation per protein may seem more straightforward when studying general physiological responses. However, incorporating multiple annotations may offer better resolution for functional annotation when investigating specific proteins. For instance, some proteins can perform multiple functions in different cell compartments and be part of more than one metabolic pathway. Nevertheless, the choice of a specific database should not solely depend on the number of annotations but also on the biological question, considering that all mentioned databases categorize proteins differently (refer to “Methods”; “Bioinformatic workflow”).

In addition to functional annotation, the pipeline which incorporated the eggNOG- mapper feature to assign the best taxonomic levels to searched protein queries, identified the taxonomic origin of transcripts and proteins. The results of the taxonomic assignment were consistent among all samples. 77–84% of functionally annotated proteins (final dataset) were assigned a best taxonomic level Bacillaryophyta (diatom dataset) (Fig. 8). On average 16% of functionally annotated proteins were assigned only to Eukaryota, 4% to Bacteria and less than 1% to Bacteria and viruses (Fig. 4). These results indicated the need for taxonomic filtering of transcriptomes, especially if transcriptomes are intended as a reference for taxonomic annotation of, for example, metatranscriptome samples. It is also important to highlight that proteins assigned to best taxonomic level Eukaryota could “hide” some diatom genes/proteins not yet annotated for diatoms, but being highly conserved among eukaryotes they were identified by eggNOG on a higher taxonomic level.

The efficacy of functional annotation of protein sequences assigned to best taxonomic level Bacillariophyta remained consistent with the final dataset. Within the diatom dataset, 75% of all proteins received at least one functional annotation, while 25% corresponded to diatom proteins of unknown function but was identified in sequenced diatom genomes. In the diatom dataset, as in the final dataset (all taxonomic levels), the majority of protein sequences received an eggNOG description (75%), followed by KO (49%) and GO terms (33%) (Supplementary Table 4). The functional annotation of diatom transcriptomes reflects the abundance of known and characterized diatom transcripts, proteins, and genes in the reference databases used for annotation. The segment of the dataset corresponding to proteins of unknown function but present in diatom genomes is yet to be experimentally characterized. Nevertheless, even without the provided annotation, these proteins have the potential to serve as a "transcriptome fingerprint" for the specific diatom culture and growth condition in future (meta)transcriptome studies. Progress in diatom transcript annotation will inevitably further improve the coverage of annotation in the NA dataset.

The diatom genera (*Pseudo-nitzchia*, *Skeletonema* and *Chaetoceros*) included in our regional transcriptome database (isolated from the northern Adriatic Sea) have been studied in previous environmental^19,48,55,84,85^ and in vitro experimental studies^19^ and their ecological importance and ability to cope with different environmental conditions has been demonstrated. Confronted with unfavourable environmental conditions phytoplankton reacts with physiological acclimation and even genetic adaptation^63^. Among other adaptation mechanisms they often remodel lipids. Some examples of this adaptation on physiological level are reported by Martin et al., where it was found that *Thalassiosira pseudonana* under P-limited condition replaces phospholipids phosphatidylcholine (PC) by the nitrogen-containing betaine lipid diacylglyceryl-carboxyhydroxymethyl-choline (DGCC) and phosphatidylglycerol (PG) by sulfolipid sulfoquinovosyldiacylglycerol (SQDG)^86^. While Abida et al., found *Phaeodactilum tricornutum* to adapt to adverse nutrient starvation conditions by replacing phospholipids with non-phospholipids^87^. These changes in environmental conditions also significantly affect protein biosynthesis and the level of phosphorylated metabolites that can affect a number of metabolic functions, including growth and the ability to photosynthesize. A high proportion of phospholipids and relatively constant composition of lipids in *Leptocylindrus* species cultured in nutrient replete and phosphate depleted conditions, suggest their evolutionary adaptation to phosphate scarcity^63^. They apparently developed mechanisms by which they take organic phosphate from the environment, as confirmed by alkaline phosphatases (AP) activity measurements. The molecular mechanisms underlying aforementioned adaptations and physiological responses still remain unclear. Understanding those mechanisms in vitro and in situ heavily relies on the availability of reference dataset like the ones described here and on the in-depth analysis of the condition-specific datasets. In addition, species-specific transcriptomes in the reference database improve the annotation of species targeted transcripts in metatranscriptomes, similar to how an additional growth condition for the species would enable the recognition of condition-specific transcripts. At the same time, the regional character of the reference database could allow to consider regional specificities of transcripts and enrich species-specific physiological patterns, gene expression and biogeographical studies.

The MMTSP database encompasses transcriptomes from 194 isolates representing 71 diatom species, originating from diverse locations across the world's oceans and cultivated under various conditions (Bioproject accession number PRJNA231566). Within the genus *Chaetoceros*, transcriptomes were available for eight distinct species, while for the genera *Pseudo-nitzchia*, *Skeletonema*, and *Thalassiosira*, transcriptomes for six species were available. Our NA dataset introduced three novel species: *Chaetoceros protuberans*, *Chaetoceros danicus,* and *Pseudo-nitzchia mannii*. Notably, for *Chaetoceros curvisetus* and *Skeletonema marinoi*, our dataset included transcriptomes specifically generated under phosphate deprived condition (P-limited), significantly expanding the available information (Tables 1 and 2). Furthermore, our dataset marked a significant milestone as the first transcriptome dataset of diatom species isolated from the Adriatic Sea and generated under environmentally relevant conditions for the area, as shown before. While the MMETSP dataset contained seven transcriptomes for *Skeletonema marinoi* isolates, two of them being from Adriatic Sea isolates (MMETSP1039 and MMETSP1040), the here presented NA dataset uniquely included transcriptomes under P-limited conditions for this species (CIM843) (Tables 1 and 2). With P-limited transcriptomes present, NA reference dataset significantly contributes to nutrient related diatom studies, since transcriptomes simulating phosphate deprived conditions in the MMETSP are scarce for species within the genera *Chaetoceros*, *Skeletonema*, *Pseudo-nitzchia*, and *Thalassiosira*. Only two species of the genus *Thalassiosira* (*T. rotula* MMETSP0912 and *T. gravida* MMETSP0494) grew under conditions (only 0.4 uM phosphate in F/2 medium) similar to the P-limited medium of NA cultures.

As highlighted in the MMETSP metadata available in the ENA archive (https://www.ebi.ac.uk/ena/browser/view/PRJNA231566), numerous researchers aimed to supply axenic and uni-algal total RNA extracts for sequencing, although this goal was not consistently achieved. This was also demonstrated by the number of non-diatom sequences retrieved in our reanalysis. Similarly, our extracts appeared to contain non-diatom DNA, prompting us to utilize the eggNOG-mapper software for functional annotation and taxonomic origin prediction. Addition of taxonomic origin prediction of genes (proteins) enabled us to differentiate the taxonomic origins of genes within our dataset. As the MMETSP transcriptomes, similar to the NA transcriptomes, did contain sequences not assigned to diatoms, we utilized the eggnog-mapper feature to determine the best taxonomic levels for proteins. The results of taxonomic assignment proved to be consistent in both MMETSP and NA datasets, with the percentage of proteins assigned to the best taxonomic level Bacillariophyta ranging from 76 to 85% in MMETSP and 77–85% in NA transcriptomes (Fig. 8). Taxonomic filtering of reference transcriptomes appears to be highly advisable, especially for samples that are not rendered axenic, particularly when treatments are expected to adversely impact diatom growth and/or transcriptional activity. When MMETSP and NA transcriptomes of each diatom species were compared, number of assembled transcripts, predicted proteins and functionally annotated proteins were similar. It appeared that the average number of predicted protein sequences varied more between the species than within different conditions for the same species. The number of predicted proteins was one order of magnitude greater for *S. marinoi* compared to *C. curvisetus*. Still, the transcriptomes *S. marinoi* MMETSP0920 and *C. curvisetus* CIM950 P-limited had notably less predicted protein sequences than all other transcriptomes. Although surprising, since these two samples had high number of assembled transcripts, the smallest average transcript lengths (606 and 350, respectively) of the two samples might explain the lower number of predicted protein sequences (Table 4). Evidence about the parameters such as the number of assembled transcripts and transcript length, and how these parameters affect the number of predicted proteins, all describe database quality. Described quality supports the usage of NA transcriptomes as a reference. We would expect that the higher the total number of transcripts and the longer the transcript, the more successful the protein prediction would be. However, the available reference studies that characterise the good quality of a transcriptome reference database are still missing and to perform a valid statistical analysis and test different parameters effect on database quality requires a more extensive transcriptome dataset for each species and growth condition than is available within NA and MMETSP. The differences between taxa in transcript lengths and total number of transcripts may be due to differences in sample quality that occur during the methodological steps of RNA isolation, library preparation and sequencing. To a certain extent, these differences are to be expected as different taxa have different morphological and physiological characteristics that could influence these methodological steps. Growth conditions could also affect expression, which is characterised by the quality and quantity of transcripts. A comprehensive benchmarking study would be needed to include and evaluate all these parameters and methodological steps for the reference database quality assessment.

Annotated proteins were for both reference datasets (MMETSP and NA) divided in two categories: those receiving at least one functional annotation (eggNOG description, KO, or GO term) and those with unknown function but previously identified in sequenced genomes. For all *S. marinoi* transcriptomes, the average percentage of proteins with at least one functional annotation was 75% (Table 5). In contrast, *C. curvisetus* transcriptomes exhibited a higher percentage, surpassing 80% in all samples (Table 5). This difference may indicate a limitation in the searched databases for functional annotation, particularly concerning the number of species with sequenced genomes. The predominant source of annotations across all samples was eggNOG description (99%), followed by KO (59–80%) and GO term (21–55%). This distribution aligns with expectations, considering that the eggNOG database (v.5) is the most comprehensive one^77,80,81^ (Table 5). The functional analysis of diatom datasets demonstrated consistency in both MMETSP and NA *C. curvisetus* and *S. marinoi* transcriptomes. Across all samples, the highest number of acquired unique annotations was observed for KO, followed by eggNOG and GO unique annotations. In addition, the distribution of proteins in COG categories in MMETSP *C. curvisetus* and *S. marinoi* transcriptomes does not exhibit notable differences from the NA dataset as well. Eventhough, MMETSP database is not the gold standard for the inclusion of new datasets in a reference database, MMETSP is nevertheless an impressively comprehensive database for microbial community research and is currently the only database of its kind available. Therefore, the comparison of the NA database with MMETSP to characterise the quality of the NA database and to point out common and/or unique features of both databases, serves as the landmark for the NA quality and supports the consistent usage of the two reference databases in the future.

## Conclusion

The regional transcriptome database generated in this study consists of 12 individual transcriptome assemblies of diatom species isolated from the northern Adriatic and grown under two conditions representative of contrasting in situ conditions with high and low phosphate availability. Those conditions in situ, generate the highest alkaline phosphatase activities measurable in marine environments^53^. NA reference transcriptomes might be used to study current environmental conditions (P-limited), particularly in the northern Adriatic, but also in global ocean environments. As for the current/present conditions, reference database provides valuable data for interpretation and understanding of future/extreme scenarios^88,89^ as well^88,89^. The NA reference database raw data obtained is publicly available in the ENA archive under accession number PRJEB74140 together with the metadata required for further data analyses. The applied bioinformatic pipeline that encompasses eggNOG-mapper feature to assign functional annotations from several different databases resulted in a high rate of successfully annotated protein sequences, in total 97,271 or 91.5%. Distinguishing proteins with annotations revealed two categories: those receiving at least one functional annotation (eggNOG description, KO, or GO term) and those with unknown function but previously identified in sequenced genomes. Annotated protein sequences may serve to further enhance our understanding of diatom physiology while the segment of the dataset corresponding to proteins of unknown function remains to be experimentally characterized. These uncategorised proteins still have the potential to serve as a "transcriptome fingerprint" for the specific diatom culture, species and growth condition in future (meta)transcriptome studies. The eggNOG-mapper feature to assign best taxonomic levels to searched protein queries, shed light on the taxonomic origin of proteins. 77–84% of functionally annotated proteins were assigned a best taxonomic level Bacillariophyta. On average 16% of functionally annotated proteins were assigned to Eukaryota, 4% to Bacteria and less than 1% to Archaea and Viruses. These results proved to be consistent when compared with the MMETSP *C. curvisetus* and *S. marinoi* datasets, highlighting the need for taxonomic filtering of transcriptomes, especially if transcriptomes are intended as a reference for taxonomic annotation of, for example, metatranscriptome samples. Nevertheless, proteins assigned to best taxonomic level Eukaryota might still “hide” some highly conserved diatom genes/proteins and therefore should not be overlooked if transcriptomes are intended for diatom physiology studies. These sequences might be particularly novel diatom sequences that our study is adding to reference databases. Comprehensive diatom transcriptome assemblies included in the analysed NA dataset have the potential to significantly expand the knowledge accumulated for these genera/species and encourage/enable further research using omics methods. All the diatoms included in our study represent species with global distribution (www.gbif.org). We hence also expect the dataset to be instrumental in future research in the region but also in future research that aims at comparing metabolic capacities of those species with a spatial component as well as research around the world that involves the aforementioned species.

### Supplementary Information

Supplementary Information.

## Data Availability

The data for this study have been deposited in the European Nucleotide Archive (ENA) at EMBL-EBI under accession number PRJEB74140.
