# Author Correction: First regional reference database of northern Adriatic diatom transcriptomes

**DOI:** 10.1038/s41598-024-69684-x

**Published:** 2024-08-12

**Authors:** Mia Knjaz, Ana Baricevic, Mirta Smodlaka Tankovic, Natasa Kuzat, Ivan Vlasicek, Lana Grizancic, Ivan Podolsak, Martin Pfannkuchen, Tjasa Kogovsek, Daniela Maric Pfannkuchen

**Affiliations:** Center for Marine Research, Ruđer Bošković Institute, Rovinj, Croatia

Correction to: *Scientific Reports* 10.1038/s41598-024-67043-4, published online 13 July 2024.

In the original version of this Article, Mirta Smodlaka Tankovic was omitted as a corresponding author. Correspondence and requests for materials should also be addressed to mirta@cim.irb.hr.

The original Article has been corrected.

