# Supplementary material for: First regional reference database of northern Adriatic diatom transcriptomes
Source: Sci Rep. 2024 Jul 13;14:16209. doi: 10.1038/s41598-024-67043-4 (PMC11246432; doi:10.1038/s41598-024-67043-4)
Supplement: Supplementary file 1 — Supplementary Information. [file 41598_2024_67043_MOESM1_ESM.docx]

**Supplementary table 1** Molecular identification details. Primer name - as defined in papers. Sequence - 5'-3' nucleotide sequence. Barcode - targeted gene. Barcode region - targeted region. Amplicon size (bp) - size of targeted ragion in base pairs. Reference - reference publication. *Zimmermann, J., Jahn, R. & Gemeinholzer, B. Barcoding diatoms: Evaluation of the V4 subregion on the 18S rRNA gene, including new primers and protocols. Org Divers Evol 11, 173–192 (2011). Hadziavdic, K. et al. Characterization of the 18s rRNA gene for designing universal eukaryote specific primers. PLoS One 9, (2014). Scholin, C. A. et al. Ribosomal DMA sequences discriminate among toxic and non‐toxic Pseudonitzschia species. Nat Toxins 2, 152–165 (1994). Bruder, K. & Medlin, L. K. Molecular assessment of phylogenetic relationships in selected species/genera in the naviculoid diatoms (Bacillariophyta). I. The genus Placoneis. Nova Hedwigia 85, 331–352 (2007). Alverson, A. J. Molecular Systematics and the Diatom Species. Protist 159, 339–353 (2008).

| **Name and nucleotide sequence** | **Barcode** | **Barcode region** | **Barcode region size (bp)** | **Reference*** |
| --- | --- | --- | --- | --- |
| D512for, 5'-ATTCCAGCTCCAATAGCG-3' | 18 S rRNA | V4 | ≈ 400 | Zimmerman et al. 2011. |
| D978rev, 5'-GACTACGATGGTATCTAATC-3' |  |  |  |  |
| F-566, 5'-CAGCAGCCGCGGTAATTCC-3' | 18 R rRNA | V4-V5 | ≈ 650 | Hadziavdic et al. 2014. |
| R-1200, 5'-CCCGTGTTGAGTCAAATTAAG -3' |  |  |  |  |
| D1R, 5'-ACCCGCTGAATTTAAGCATA-3' | 28 S rRNA | D1-D3 | ≈ 800 | Scholin et al. 1994., Bruder & Medlin 2007. |
| D3Ca, 5'-ACGAACGATTTGCACGTCAG-3' |  |  |  |  |
| rbcL66+,  5'-TTAAGGAGAAATAAATGTCTCAATCTG-3' | rbcL | 5’ end region | ≈ 800 | Alverson 2008., MacGillivary & Kaczmarska 2011. |
| DtrbcL3R, 5'-ACACCWGACATACGCATCCA-3' |  |  |  |  |

**Supplementary table 2** Bioinformatic workflow details. *Number of predicted protein sequences corresponds to number of proteins before the exclusion of proteins corresponding to transcripts with zero counts.

|  |  |  | **Seq** | **SortMeRNA** | | **Trimmomatic** | **Trinity** | **TransDecoder** |
| --- | --- | --- | --- | --- | --- | --- | --- | --- |
| **Culture ID** | **Species** | **Condition** | **Number of reads** | **Number of rRNA reads** | **Number of mRNA reads** | **Number of quality trimmed reads** | **Number of transcripts** | **Number of predicted protein sequences*** |
| CIM827 | *Chaetoceros protuberans* | F/2 | 43,869,248 | 6,221,316 | 36,808,162 | 35,892,984 | 4,204 | 2,927 |
|  |  | P-limit | 46,060,370 | 24,998,485 | 20,053,033 | 19,291,546 | 55,259 | 25,955 |
| CIM843 | *Skeletonema marinoi* | F/2 | 47,142,298 | 1,324,312 | 44,888,944 | 43,905,102 | 64,544 | 41,609 |
|  |  | P-limit | 46,959,304 | 8,074,922 | 37,955,062 | 36,962,568 | 70,298 | 38,335 |
| CIM950 | *Chaetoceros curvisetus* | F/2 | 45,113,058 | 8,197,533 | 35,893,711 | 34,955,780 | 50,374 | 31,042 |
|  |  | P-limit | 35,562,540 | 26,151,030 | 8,259,394 | 7,855,370 | 22,886 | 6,612 |
| CIM964 | *Chaetoceros danicus* | F/2 | 41,065,412 | 2,303,237 | 37,714,375 | 36,587,302 | 75,087 | 46,568 |
|  |  | P-limit | 35,182,380 | 9,693,592 | 24,478,642 | 23,446,778 | 63,106 | 39,874 |
| CIM  1008 | *Pseudo-nitzschia mannii* | F/2 | 46,749,028 | 8,225,002 | 37,602,452 | 36,708,516 | 52,978 | 27,183 |
|  |  | P-limit | 43,417,630 | 25,684,530 | 16,731,692 | 16,183,690 | 34,986 | 16,378 |
| CIM  1063 | *Thalassiosira sp.* | F/2 | 38,814,556 | 8,032,436 | 30,019,372 | 29,241,382 | 108,010 | 41,739 |
|  |  | P-limit | 49,200,702 | 1,907,837 | 45,610,305 | 44,147,886 | 177,452 | 104,752 |

**Supplementary table 3** Overview of the final dataset characteristics (sequences abundance) after eggNOG functional annotation. eggNOG description – number of protein sequences annotated with eggNOG description. KOs – number of protein sequences annotated with KO term. GOs – number of protein sequences annotated with GO term.

|  |  |  | **Functional annotations of final dataset (all taxonimic levels)** | | |
| --- | --- | --- | --- | --- | --- |
| **Culture ID** | **Species** | **Condition** | **eggNOG description** | **KOs** | **GOs** |
| CIM827 | *Chaetoceros_protuberans* | F/2 | 703 | 465 | 306 |
|  |  | P-limit | 4,972 | 3,515 | 2,381 |
| CIM843 | *Skeletonema_marinoi* | F/2 | 11,214 | 6,779 | 4,183 |
|  |  | P-limit | 7,561 | 4,752 | 2,987 |
| CIM950 | *Chaetoceros_curvisetus* | F/2 | 7,112 | 4,770 | 3,043 |
|  |  | P-limit | 790 | 639 | 443 |
| CIM964 | *Chaetoceros_danicus* | F/2 | 8,723 | 5,759 | 3,598 |
|  |  | P-limit | 6,171 | 4,079 | 2,567 |
| CIM1008 | *Pseudo-nitzschia mannii* | F/2 | 5,962 | 3,976 | 2,525 |
|  |  | P-limit | 2,337 | 1,591 | 1,033 |
| CIM1063 | *Thalassiosira sp.* | F/2 | 3,985 | 2,502 | 1,583 |
|  |  | P-limit | 15,658 | 9,160 | 5,432 |

**Supplementary table 4** Overview of the diatom dataset characteristics (sequences abundance) after eggNOG functional annotation. eggNOG description – number of protein sequences and unigenes annotated with eggNOG description. KOs – number of protein sequences and unigenes annotated with KO term. GOs – number of protein sequences and unigenes annotated with GO term.

|  |  |  | **Functional annotations of diatom dataset (taxonomic level Bacillariophyta)** | | | **Bacillariophyta unique proteins (unigenes)** | | |
| --- | --- | --- | --- | --- | --- | --- | --- | --- |
| **Culture ID** | **Species** | **Condition** | **eggNOG description** | **KOs** | **GOs** | **eggNOG description** | **KOs** | **GOs** |
| **CIM827** | ***Chaetoceros protuberans*** | F/2 | 564 | 398 | 274 | 409 | 313 | 223 |
|  |  | P-limit | 3,873 | 2,803 | 1,993 | 1,846 | 1,882 | 1,458 |
| **CIM843** | ***Skeletonema marinoi*** | F/2 | 9,104 | 5,745 | 3,701 | 2,698 | 2,869 | 2,179 |
|  |  | P-limit | 6,103 | 3,979 | 2,638 | 2,348 | 2,429 | 1,827 |
| **CIM950** | ***Chaetoceros curvisetus*** | F/2 | 5,407 | 3,744 | 2,480 | 2,228 | 2,319 | 1,761 |
|  |  | P-limit | 601 | 503 | 360 | 461 | 432 | 323 |
| **CIM964** | ***Chaetoceros danicus*** | F/2 | 6,619 | 4,474 | 2,972 | 2,308 | 2,461 | 1,869 |
|  |  | P-limit | 4,698 | 3,186 | 2,126 | 1,974 | 1,980 | 1,478 |
| **CIM1008** | ***Pseudo-nitzschia mannii*** | F/2 | 4,535 | 3,112 | 2,052 | 1,939 | 1,998 | 1,484 |
|  |  | P-limit | 1,836 | 1,297 | 888 | 1,115 | 1,034 | 754 |
| **CIM1063** | ***Thalassiosira sp.*** | F/2 | 3,168 | 2,055 | 1,405 | 1,476 | 1,383 | 1,019 |
|  |  | P-limit | 11,787 | 7,157 | 4,581 | 2,656 | 2,826 | 2,149 |

**Supplementary table 5** Overview of the diatom assigned proteins (sequences abundance) after eggNOG functional annotation and best taxonomic level prediction of MMETSP and NA C. curvisetus and S. marinoi transcriptomes. Bacillariophyta – number of protein sequences assigned a best taxonomic level Bacillaryophyta (diatoms). eggNOG description – number of unigenes based on eggNOG description annotations. KOs – number of unigenes based on KO term annotations. GOs – number of unigenes based on GO term annotations.

|  |  |  | **eggNOG best taxonomic level** | **Bacillariophyta unique proteins (unigenes)** | | |
| --- | --- | --- | --- | --- | --- | --- |
| **Culture ID** | **Species** | **Condition** | **Bacillariophyta** | **eggNOG description** | **KOs** | **GOs** |
| MMETSP0319 | *Skeletonema marinoi* | F/2 (-Si -Cu) | 14,374 | 2,690 | 2,847 | 1,942 |
| MMETSP0320 | *Skeletonema marinoi* | F/2 | 14,830 | 2,717 | 2,898 | 1,987 |
| MMETSP0918 | *Skeletonema marinoi* | F/2 | 15,635 | 2,798 | 2,993 | 2,067 |
| MMETSP0920 | *Skeletonema marinoi* | F/2 -Si | 349 | 166 | 116 | 64 |
| MMETSP1039 | *Skeletonema marinoi* | F/2 -light | 8,705 | 1,941 | 1,889 | 1,268 |
| MMETSP1040 | *Skeletonema marinoi* | F/2 +light | 8,623 | 1,871 | 1,880 | 1,242 |
| MMETSP1428 | *Skeletonema marinoi* | Standard Aquil | 15,595 | 2,731 | 2,916 | 2,021 |
| CIM843 | *Skeletonema marinoi* | F/2 | 12,658 | 2,698 | 2,869 | 2,179 |
| CIM843 | *Skeletonema marinoi* | P-limit | 8,391 | 2,348 | 2,429 | 1,827 |
| MMETSP0718 | *Chaetoceros curvisetus* | ASW -NO3 | 3,335 | 1,386 | 1,339 | 908 |
| MMETSP0719 | *Chaetoceros curvisetus* | ASW +nocodazole | 35 | 30 | 26 | 21 |
| CIM950 | *Chaetoceros curvisetus* | F/2 | 6,895 | 2,228 | 2,319 | 1,761 |
| CIM950 | *Chaetoceros curvisetus* | P-limit | 684 | 461 | 432 | 323 |
